# Electroacupuncture Promotes Remyelination after Cuprizone Treatment by Enhancing Myelin Debris Clearance

**DOI:** 10.3389/fnins.2016.00613

**Published:** 2017-01-10

**Authors:** Keying Zhu, Jingxian Sun, Zheng Kang, Zaofeng Zou, Gencheng Wu, Jun Wang

**Affiliations:** ^1^Department of Integrative Medicine and Neurobiology, School of Basic Medical Sciences, Shanghai Medical College, Fudan UniversityShanghai, China; ^2^State Key Laboratory of Medical Neurobiology, Collaborative Innovation Center for Brain Science, Institutes of Brain Science, Fudan UniversityShanghai, China; ^3^Academy of Integrative Medicine, Fudan UniversityShanghai, China

**Keywords:** cuprizone, remyelination, electroacupuncture, microglia, multiple sclerosis, myelin debris

## Abstract

Promoting remyelination is crucial for patients with demyelinating diseases including multiple sclerosis. However, it is still a circuitous conundrum finding a practical remyelinating therapy. Electroacupuncture (EA), originating from traditional Chinese medicine (TCM), has been widely used to treat CNS diseases all over the world, but the role of EA in demyelinating diseases is barely known. In this study, we examined the remyelinating properties and mechanisms of EA in cuprizone-induced demyelinating model, a CNS demyelinating murine model of multiple sclerosis. By feeding C57BL/6 mice with chow containing 0.2% cuprizone for 5 weeks, we successfully induce demyelination as proved by weight change, beam test, pole test, histomorphology, and Western Blot. EA treatment significantly improves the neurobehavioral performance at week 7 (2 weeks after withdrawing cuprizone chow). RNA-seq and RT-PCR results reveal up-regulated expression of myelin-related genes, and the expression of myelin associated protein (MBP, CNPase, and O4) are also increased after EA treatment, indicating therapeutic effect of EA on cuprizone model. It is widely acknowledged that microglia exert phagocytic effect on degraded myelin debris and clear these detrimental debris, which is a necessary process for subsequent remyelination. We found the remyelinating effect of EA is associated with enhanced clearance of degraded myelin debris as detected by dMBP staining and red oil O staining. Our further studies suggest that more microglia assemble in demyelinating area (corpus callosum) during the process of EA treatment, and cells inside corpus callosum are mostly in a plump, ameboid, and phagocytic shape, quite different from the ramified cells outside corpus callosum. RNA-seq result also unravels that most genes relating to positive regulation of phagocytosis (GO:0050766) are up-regulated, indicating enhanced phagocytic process after EA treatment. During the process of myelin debris clearance, microglia tend to change their phenotype toward M2 phenotype. Thus, we also probed into the phenotype of microglia in our study. Immuno-staining results show increased expression of CD206 and Arg1, and the ratio of CD206/CD16/32 are also higher in EA group. In conclusion, these results demonstrate for the first time that EA enhances myelin debris removal from activated microglia after demyelination, and promotes remyelination.

## Introduction

Multiple sclerosis (MS) is an autoimmune-mediated demyelinating disease of central nervous system (CNS), with a hallmark of extensive demyelination in the CNS. Though clinically, there is no ideal treatment for MS hitherto, disease modifying therapies (DMTs) are the mainstream for MS treatment (Oh and O'Connor, 2015). Currently approved DMTs are immunosuppressive and immunomodulatory agents. These agents help prevent disease relapse and reduce the severity of relapse to some extent (Cross and Naismith, 2014; Wingerchuk and Carter, 2014). However, DMTs can only partially postpone the relapse of MS and slightly reduce the accumulation of physical disabilities. Besides, high cost and side effect of DMT is also unfavorable for MS patients (Adelman et al., 2013; Carrithers, 2014; Winkelmann et al., 2015). The process of remyelination is directly and closely related to the restoration of neuronal function and the amelioration of clinical disabilities (Nave, 2010; Harlow et al., 2015; Olsen and Akirav, 2015). However, DMTs have limited impact on remyelination. So far, no practical remyelinating therapy has been applied clinically and nearly all potential remyelinating methods are still under development (Hartley et al., 2014).

Acupuncture as well as electroacupuncture (EA), a therapeutic intervention originating from traditional Chinese medicine (TCM), has long been used to treat various disorders (Wootton, 1997). Compelling evidence demonstrates that acupuncture is beneficial in preventing neuronal injury in various pathological conditions, such as stroke, cerebral palsy, depression, etc (Han and Ho, 2011; Kwon et al., 2012; Xu et al., 2013; Bai et al., 2014; MacPherson et al., 2014; Yang et al., 2014). Clinical reports have yielded promising evidence of acupuncture ameliorating symptoms of MS patients, such as fatigue, bladder dysfunction, spasticity, and eventually improving quality of life (Tjon et al., 2009; Kopsky and Hesselink, 2012; Quispe-Cabanillas et al., 2012; Foroughipour et al., 2013). In addition, Laboratory studies based on experimental autoimmune encephalomyelitis (EAE) model, a classic animal model mainly mimicking the immunopathology of MS, also show that EA treatment inhibit the proliferation of encephalitogenic T cells, increase the secretion of ACTH and β-endorphin, and eventually ameliorate EAE (Park et al., 2004; Liu et al., 2013). A recent study indicates that EA promotes remyelination in compressed spinal cord injury (SCI) model by enhancing the proliferation of oligodendrocyte precursor cells (Huang et al., 2015). Additionally, EA promotes the differentiation of the transplanted bone marrow mesenchymal stem cells into oligodendrocyte-like cells in SCI model (Ding et al., 2013; Liu et al., 2015).

SCI model is a traumatic local demyelinating model rather than a systematic demyelinating model (Ransohoff, 2012; Procaccini et al., 2015). Hence, we introduce cuprizone (CPZ)-induced demyelinating model in our study. CPZ-induced demyelinating model is a classic rodent model allowing the investigation specifically in CNS demyelination and remyelination (Kipp et al., 2009; Zendedel et al., 2013; Praet et al., 2014). By feeding mice with chow containing 0.2% CPZ for 5 weeks, significant CNS demyelination can be induced, with corpus callosum (CC) being the most vulnerable region (Steelman et al., 2012; Praet et al., 2014). After removal of CPZ from diet, spontaneous remyelination occurs over time. Therefore, CPZ model is a systematic model for the study of demyelination and remyelination of MS. It is unclear whether EA is effective in CPZ model and the underlying mechanisms of EA in promoting remyelination remain unraveled.

Previous studies have confirmed that CPZ-induced demyelination is accompanied with extensive accumulation of degraded myelin debris and activation of glial cells, chiefly CNS microglia and astrocytes (Praet et al., 2014). Microglia is verified to play an important role in the process of remyelination (Voss et al., 2012; Miron et al., 2013; Doring et al., 2015; Lampron et al., 2015). Accumulating evidence suggests that microglia is associated with the clearance and phagocytosis of degraded and collapsed myelin debris, existence of which is detrimental to OPC proliferation and remyelination (Kotter et al., 2005, 2006; Ruckh et al., 2012; Kocur et al., 2015). Studies also found that microglia, especially M2 phenotype, are positively related to the recruitment of OPCs and their differentiation into mature oligodendrocytes, as well as myelin formation (Miron et al., 2013; Wang et al., 2015; Marteyn et al., 2016). Although EA shows a therapeutic effect on various neurodegenerative diseases achieved by its ability to alleviate existing neuroinflammation and glial dysfunction, it is unclear whether EA could modulate microglia function during the process of demyelination and remyelination.

The current studies were performed to understand the therapeutic effect and potential mechanisms of EA in CPZ-induced demyelinating model. We assessed the neurobehaviors in CPZ fed and EA treated mice and the expression of myelin associated markers, and probed into the mechanisms by assessing the production of degraded myelin debris and the recruitment of microglia into CC as well as the phenotype of microglia in CC. To our knowledge, this is the first study ever focusing on the function of EA in CPZ-induced demyelinating model and the regulatory effect of EA on microglia in demyelinating diseases.

## Materials and methods

### Animals and CPZ feeding scheme

Male C57BL/6 mice aged between 5 and 6 weeks, acquired from the Experimental Animal Center, Chinese Academy of Sciences (Shanghai, China), were used in the present study. To induce demyelination, mice were fed with standard rodent chow containing 0.2% CPZ powder (Sigma-Aldrich, St. Louis, MO, USA) for 5 weeks. After 5 weeks' induction, CPZ was removed and replaced with standard diet for 2 weeks allowing for spontaneous remyelination. All protocols were performed and approved in accordance with the National Institutes of Health Guide for the Care and the Animal Research Welfare Council of School of Basic Medical Science of Fudan University (20140266-086). All endeavors were made as far as possible to reduce the sacrifice of animals and relieve their sufferings during the experiments.

### Electroacupuncture (EA) treatment

EA treatment started from the first day of week 5 and lasted for 3 weeks. Mice received 30 min EA treatment once every other day. Two acupoints in the governor vessel (GV) were adopted, which is Baihui (GV20) and Zhiyang (GV9). A pair of stainless needles with diameters of 0.3 mm (Suzhou Medical Supplies, Suzhou, P.R. of China) were obliquely and subcutaneously inserted into GV9 and GV20 to a depth of 5 mm or so but exterior to the harnpan and vertebral canal. The pair of needles were then connected with an output terminal of an EA apparatus (HANS Acupoint Nerve Stimulator, LH202H, Beijing, P.R. of China), with alternating strains of dense-sparse frequencies of 2/15 Hz and stimulating current of 3–4 mA. Mice were restrained by an apparatus made by our lab specially for the EA treatment. All other mice groups have been restrained the same way. In our preliminary study, we have set up a group of sham-EA. Mice in sham-EA group received the same acupuncture treatment without electric stimulation.

### Beam walking test

The assessment of mice locomotor coordination was performed by beam walking test (Skripuletz et al., 2010). In brief, mice were trained to traverse a 1.5 cm narrow wood beam to reach a so-called safety box with paddings inside, which created a relatively safe environment encouraging mice to traverse the beam. Mice were placed on one end of the 100 cm long beam (horizontally 60 cm above the platform), and the traversing times were recorded. To reduce errors, the time mice spent on both ends (10 cm of each end) were neglected, only the time spent in the middle 80 cm of the beam were recorded. As displayed in Video 1, we started recording at the moment hind limbs of mice passing the START line on the beam, and end recording at the moment fore limbs of mice touching the END line on the beam. Mice received two consecutive trials in each test and the mean time of two trials was used for statistical analysis (cut-off time 60 s). Thick cotton cushions were placed under the beam in case mice slip down.

### Pole test

Pole test was performed according to previous reports with minor adjustments (Lin et al., 2013). In brief, mice were placed tenderly head-up facing the apex of a vertical round wood pole (diameter: 1 cm; height: 50 cm) with gauze-wrapped rough surface enabling mice to grab. The time mice climbing over the apex of pole with head facing down and body in a vertical position (turn-back time), and the time mice descending to the bottom of the pole after climbing over (touch-down time) were recorded for analysis (Video 3). The cut-off time was 30 and 60 s for turn-back time and touch-down time, respectively. In each test, each mouse conducted two consecutive trials and the average time of two trials was recorded for statistical analysis. Thick cotton cushions were placed under the pole in case mice fall off.

### Histological analysis

Mice were gently anesthetized with saline solution of pentobarbital sodium (70 mg/kg, i.p.) and perfused with 4% paraformaldehyde. The cerebra were resected and serial 4 μm paraffin sections were stained with hematoxylin and eosins (HE) or luxol fast blue (LFB) to assess demyelination within the area of CC. For HE staining, the process was conducted following our previous protocol (Wang et al., 2012). For LFB staining, paraffin tissues were stained in LFB/cresyl violet overnight at 55°C, followed by washing in 95% ethanol and double-distilled water to remove redundant dye. Then, the white matter of the sections was distinguished from the gray matter in a lithium carbonate solution for about 15 s (until easily distinguishable). Later on, the sections were washed by double-distilled water and 75% ethanol.

### Red oil O staining

After drying in 100% propylene glycol, brain tissues were stained with 0.5% Red Oil O solution (Sigma, USA) at a temperature of 60°C for 6 min. After that the brain slices were incubated with 85% propylene glycol for 2 min following by rinsing. Nuclei were stained with haema-toxylin (Sigma, USA).

### Immunofluorescence

Paraffin sections (4 μm) were used for MBP (1:200, Millipore, AB980) and dMBP (1:2000, Millipore, AB5864) staining to determine the degree of myelination and the production of degraded myelin debris respectively. Frozen sections (25 μm) were prepared for staining with Iba1 (1:400, Wako, Japan), Arg1 (1:200, Santa Cruz, USA), iNOS (1:200, Santa Cruz, USA), CD206 (1:100, R&D, USA), CD16/32 (1:500, BD, USA), and O4 (1:50, Millipore, USA) to determine the recruitment of microglia into the area of CC and the phenotype of the recruited microglia. The sections were first washed three times and blocked with 4% goat serum in 0.3% Triton X-100 for 3 h at room temperature followed by incubation with antibodies described above at 4°C for 12 h followed by Alexa Fluor 488 or 555 goat anti-rabbit secondary antibodies (1:1000, Invitrogen, USA) at 37°C. All sections were treated with Fluorescence Decay Resistant Medium with DAPI before being covered with coverslip. The images were captured by a multiphoton laser scanning confocal microscopy system (Olympus Fluoview FV1000) or common fluorescence microscope (Leica DMI6000).

### Western blot analysis

Western blot was performed to quantify the expression of MBP. The brain was removed and CC tissue was quickly resected on ice. The CC was ultrasonically homogenized in radioimmunoprecipitation assay lysis buffer (RIPA buffer, Beyotime, Shanghai, China) followed by 12,000 rpm centrifugation for 10 min and the supernatant was collected for western blot analysis. Equal amounts of protein samples (20 μg of total protein) were analyzed by SDS-PAGE with primary antibodies being either rabbit anti-MBP (1:500, Millipore, AB980) or anti-GAPDH (1:10000, Proteintech, HRP-60004). Proteins were detected via incubation with horseradish peroxidase-conjugated secondary antibodies and an ECL chemiluminescence detection system (Tanon, Shanghai, China). The images were obtained using an ImageQuant LAS4000 mini image analyzer (GE Healthcare, Buckinghamshire, UK).

### RNA sequencing (RNA-seq) analysis

Total RNA was prepared using the Qiagen RNeasy kit. Libraries were prepared using the NEBNext Library Prep Kit (New England Biolabs) according to the manufacturer's instructions. Library quality was assessed using a Bioanalyzer (Agilent) and then the samples were sequenced on the Illumina Hiseq 2000 with a goal of 30 million reads per sample. Raw FASTQ files were aligned using PRADA and FPKM values obtained using Cuffllinks for gene expression analysis.

### Quantitative RT-PCT (qRT-PCR) analysis

To evaluate the mRNA expression of genes related to oligodendrocyte lineage and myelination, the corpus callosum were dissected 7 weeks after the first day of cuprizone feeding; total RNA were isolated using Trizol reagent (Invitrogen, USA). The relative abundance of target mRNAs were then quantified using SYBR Green qRT-PCR detection (Light Cycler 96 real-time PCR detection system, Roche, Switzerland). The primer sequences of target mRNA are listed in Table 1. The housekeeping gene, HPRT, was used as an internal reference for standardization of the analysis. Relative quantification was performed by determination of the n-fold differential expression with the 2^−ΔΔCt^ method and is expressed as relative fold change compared to HPRT. Melting curves were used to establish the purity of the amplified band. The PCR products were sequenced to confirm identity.

**Table 1:** **Primers used for RT-PCR analysis**.

**Genes**	**Species**	**Primers (5′–3′)**
MBP	Mouse	FW:AAGTACCTGGCCACAGCAAG
		RE:AGCTTCTCTACGGCTCGGA
MAG	Mouse	FW:TCTCTACCCGGGATTGTCACT
		RE:CGGATTTCTGCATACTCAGCCA
CNP	Mouse	FW:AGAGTGATCCTTGGAGCCAGA
		RE:CGGAGGGGAATGGTGGATTT
PLP1	Mouse	FW:CTGAGCGCAACGTTTGTGG
		RE:TACATTCTGGCATCAGCGCA

### Image analysis

Western blot bands and immunofluorescence staining (integrated optical density, IOD) were analyzed using ImageJ software. The quantitative statistical graphs were created based on the relative fold change.

### Statistical analysis

All quantitative data were presented as mean ± standard error of the mean (S.E.M.). Statistical analysis and graphs were obtained using Graphpad 5.0 software. Differences between groups were analyzed with Student's *t*-test and one-way analysis of variance (ANOVA) followed by LSD post-test or Bonferroni post-test. Values of *P* < 0.05 were regarded as the criteria of significance.

## Results

### CPZ administration induces neurobehavioral defects and severe demyelination

**Figure 1:**
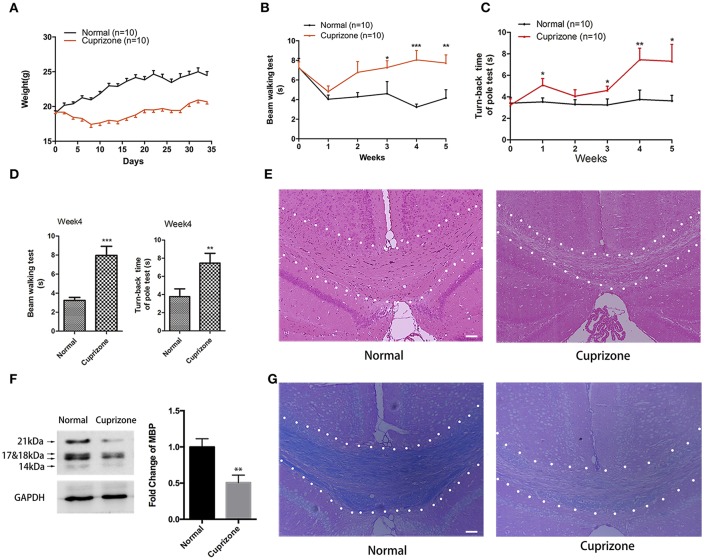
**CPZ administration induces severe demyelinating related changes**. Mice were fed with chow containing 0.2% CPZ powder for 5 weeks. **(A)** Consecutive body weight change during 5 weeks (*n* = 10 for both group). **(B)** Reverse time of beam walking test and **(C)** turn-back time of pole test in CPZ fed mice (*n* = 10 for both groups). **(D)** CPZ fed mice exhibited the most detectable defects in neurobehaviors at week 4. Pathological changes of demyelination were assessed by **(E)** HE staining and **(G)** LFB staining after 5 weeks of CPZ administration (scale bar = 50 μm). **(F)** The expression of MBP were detected by Western Blot (left), and the quantitative data of MBP expression were displayed as relative fold change compared to normal group (right, *n* = 4/group). All data represents the means ± S.E.M. (^*^p < 0.05, ^**^p < 0.01, ^***^p < 0.001 compared to normal group).

During the first 10 days after CPZ administration, we observed obvious weight loss of CPZ fed mice compared to mice with normal diet (Figure 1A). Two weeks later, CPZ fed mice gradually regained weight, but still did not weigh as much as the normal group. CPZ feeding can lead to extensive demyelination in many regions of brain including hippocampus, cerebellum, cortex, and with corpus callosum (CC) the most vulnerable one, which results in motor coordination impairment. To evaluate the motor coordinative function, beam walking test and pole test were performed. Mice exposed to CPZ exhibited severe coordinative locomotor dysfunction with the peak at and around week 4 (Figures 1B–D; Videos 1–4). As shown in the videos, in beam test CPZ fed mice tended to be more hesitant traversing the beam with more fumbles and pauses; in pole test, CPZ fed mice had more difficulty in climbing over the apex of the pole. However, the touch-down time in pole test was not affected by CPZ (Data sheet 1, Figure S1). No mice fell off or slipped over from the beam or pole during our behavioral tests. CC is previously reported as the most vulnerable region during CPZ treatment (Steelman et al., 2012). Thus, we evaluated the histomorphological change of CC region by HE and LFB staining and observed conspicuous demyelination in CC after CPZ treatment for 5 weeks (Figures 1E,G). Myelin basic protein (MBP) is the main component of oligodendrocyte as well as myelin within CC. Therefore, we further detected the amount of MBP in CC by western blot and verified obvious decrease of MBP in CPZ fed mice (Figure 1F). Those results clearly indicate a successful establishment of CPZ induced demyelinating model.

### EA modulates neurobehavioral dysfunction of demyelinating mice

**Figure 2:**
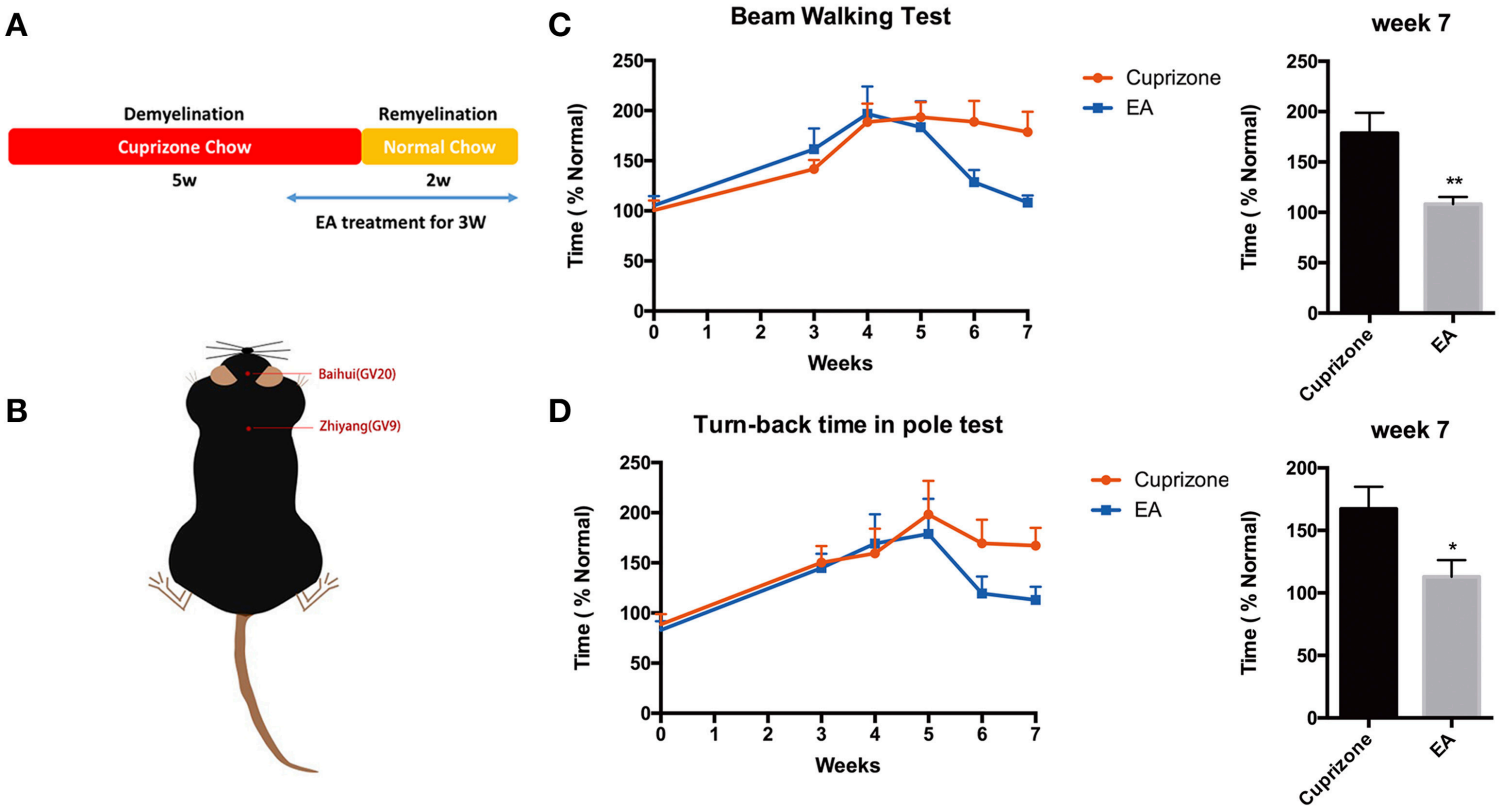
**Amelioration of neurological behaviors after 3 weeks of EA treatment**. **(A)** The scheme of experiment: CPZ administration was withdrawn after 5 weeks, followed by 2 weeks of remyelination until mice were sacrificed. Mice received 3 weeks of EA treatment since the 1st day at week 5, when they started exhibiting severe defects in neurological behaviors. **(B)** The location of Baihui (GV20) and Zhiyang (GV9) acupoints; EA treatment significantly reduced the time spent in **(C)** beam walking test and **(D)** turn-back time in pole test at week 7. Data represents the means ± S.E.M. (*n* = 10–11/group, ^*^p < 0.05, ^**^p < 0.01).

To examine the effect of EA on the neurological behaviors of mice with demyelination, we performed beam walking test and pole test, which aim to examine motor coordinative function in association with ataxia, which is one of the main clinical features of MS patients. Previously, we compared the effect of EA treatment and sham-EA treatment (described in Section Materials and Methods), and our preliminary results indicate that sham-EA treatment exert little effect on remyelination (Data sheet 1, Figure S2). Thus, to reduce unnecessary sacrifice of mice under the consideration of animal welfare, we excluded sham-EA group for the following experiments. EA treatment started at the beginning of week 5 and lasted for 3 weeks until mice were sacrificed at the end of week 7 (Figure 2A). Baihui (GV20) and Zhiyang (GV9) acupoints were used for EA treatment (Figure 2B). Mice receiving EA treatment exhibited a better performance in beam walking test (Figure 2C) and turn-back time in pole test (Figure 2D). Two weeks EA administration already effectively decreased the traverse time in beam walking test (Figure 2C); and the traverse time in beam test and turn-back time in pole test completely reverted back to normal by the end of the treatment. However, the touch-down time in pole test, which mainly indicating the muscular strength of forelimbs, was not affected either by CPZ (Figure S1) or EA treatment (data not shown). These results prompt us to conclude that EA modulates neurobehavioral dysfunction associated with demyelination, and that muscle strength of forelimbs was not affected either by CPZ or EA treatment.

### EA promotes remyelination in corpus callosum

**Figure 3:**
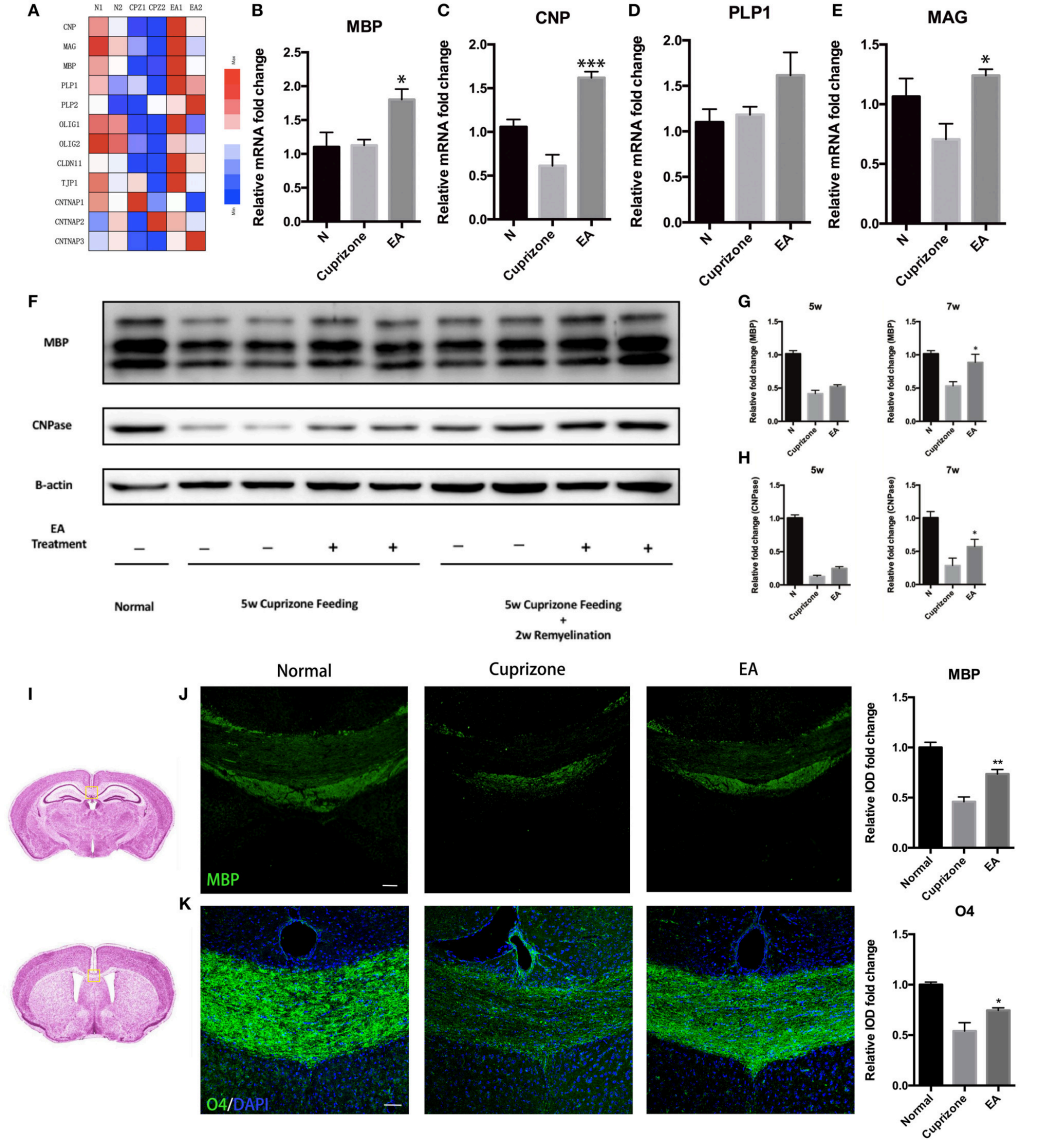
**EA promotes remyelination in corpus callosum. (A)** Heatmap shows RNA-Seq results of gene expression in corpus callosum at week 7. **(B–E)** mRNA level of genes of oligodendrocyte lineage (*MBP, CNP, PLP1, MAG*) in each group at week 7 (*n* = 4 for each group). **(F)** The protein expression of MBP and CNPase in each group at week 5 and week 7. **(G,H)** Bar graph shows the quantification of MBP and CNPase expression in **(F)** (*n* = 4 for each group). **(I)** The area of interest within the corpus callosum (yellow frame). The brain altas of mice is a pubic data from Harvard University (http://www.hms.harvard.edu/research/brain/atlas.html). The upper section (Label 305 of the atlas) is for MBP staining, and the lower section is for O4 staining. **(J,K)** Immunostaining of MBP and O4 showing the structure of myelin sheath (*n* = 4 for each group; scale bar = 50 μm). All data represents the means ± S.E.M. (^*^p < 0.05, ^**^p < 0.01, ^***^p < 0.001 compared to normal group).

CC is the most evident and notable region of demyelination in CPZ model. Thus, to evaluate the effect of EA on remyelination, we applied RNA-seq to the transcriptome of CC tissue from normal mice, CPZ fed mice and CPZ + EA treated mice at the end of week 7. We focused on the expression profiles of genes relating to myelin components, nodal/paranodal proteins, and some transcription factors that play a role in oligodendrocyte development. The RNA-seq results (Data sheet 2) revealed remarkably reduced expression of most genes in CPZ group, while EA treatment reversed the reduction and stimulated the expression of most of those myelin-related genes, notably CNP, PLP1/2, CLDN11, and CNTNAP3 (Figure 3A). To further confirm the results, we applied RT-PCR experiments to detect the mRNA expression of genes encoding proteins of myelin components and the results were basically in accordance with RNA-seq results (Figures 3B–E). Myelin basic protein (MBP) is the main component of myelin sheath, and the anti-MBP antibody is one of the most commonly used markers to examine the amount of myelin and the integrity of an intact myelin structure; CNPase is also a myelin protein which is exclusively expressed by lineage of oligodendrocytes in the CNS. To further verify the efficacy of EA in remyelination, we examined the expression of MBP and CNPase in CC by using western blot. At week 5, we did not observe obvious difference in the expression of MBP or CNPase between CPZ group and EA group; however, EA treatment for 3 weeks caused significantly increased protein expression of MBP and CNPase in CC region compared to CPZ group (Figures 3F–H). Similar results were also obtained from immunofluorescence experiments. Since demyelination in corpus callosum is uneven (more severe in caudal part), we detected demyelination in both caudal and rostral part of corpus callosum (Figure 3I) with MBP and O4. At week 7, CPZ fed mice showed decreased myelin integrity compared to normal mice as detected by MBP and O4 staining, whereas EA can significantly enhance the expression of MBP and O4 and rebuild dense myelin sheaths (Figures 3J,K). Taken together, these results confirm the efficacy of EA on remyelination.

### EA promotes the clearance of degraded myelin debris

**Figure 4:**
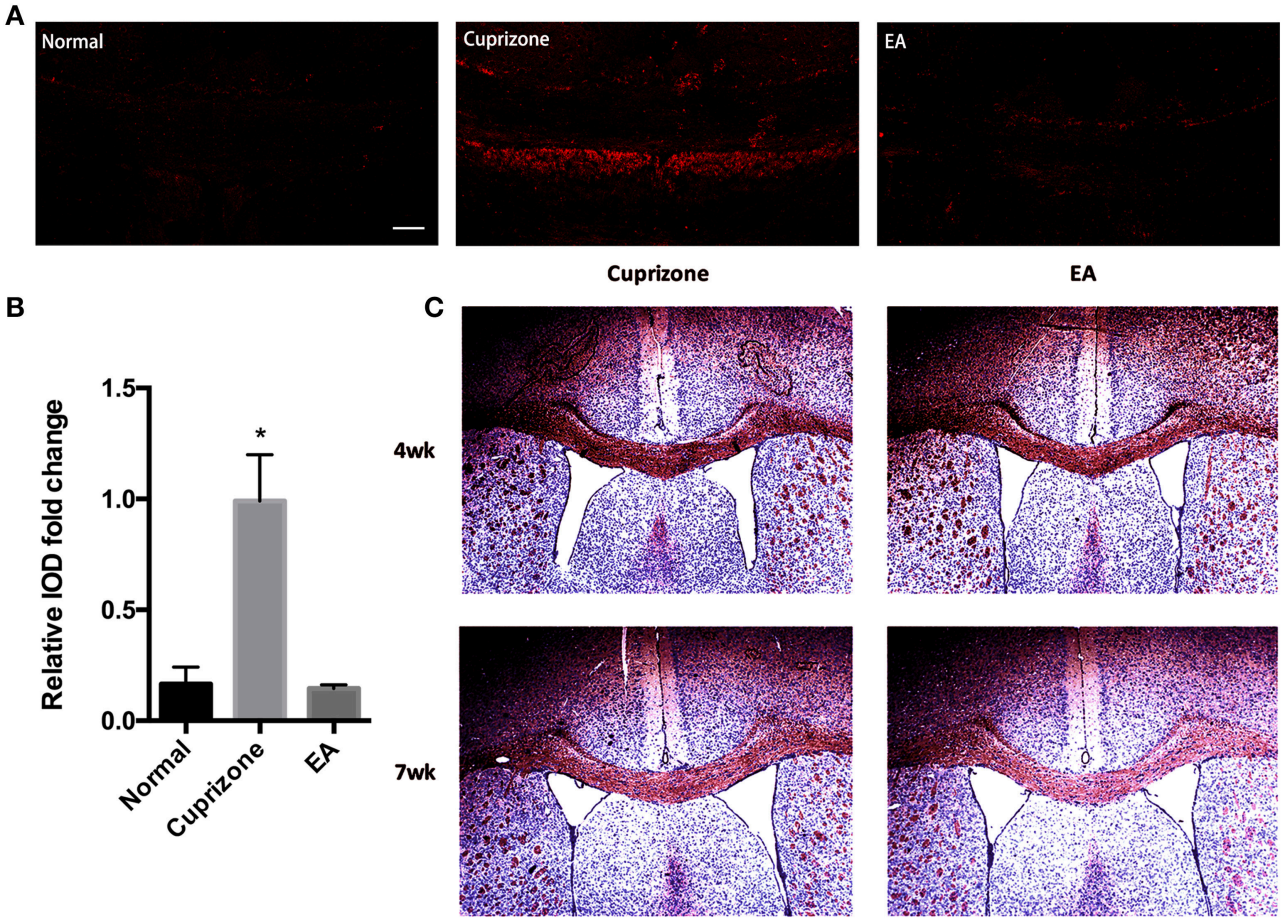
**EA treatment enhances the clearance of degraded myelin debris. (A)** The immunofluorescence staining of degenerated myelin debris was detected using dMBP antibody (red) using a confocal microscope (scale bar = 50 μm). **(B)** Bar graph shows the semi-quantification of integrated optical density (IOD) for dMBP expression. **(C)** Red oil O staining showing lipid-associated deposit of degraded myelin debris. Data represents the means ± S.E.M. (*n* = 3–4/group, ^*^p < 0.05 compared to normal and EA group).

In CPZ demyelinating model, demyelination is followed by accumulation of degraded myelin debris and activation of glial cells (Praet et al., 2014). It is reported that the accumulation of myelin debris is detrimental to CNS axonal remyelination by inhibiting the differentiation of OPC (Kotter et al., 2006). Thus, the removal and clearance of degraded myelin debris, generated during the process of demyelination, is a critical step for remyelination process (Lampron et al., 2015). To test whether the effect of EA on remyelination is related to the clearance of myelin debris, an antibody which specifically binds degraded myelin basic protein (dMBP) was used. This dMBP antibody is an important tool to study myelin debris, which has previously been reported to bond a certain MBP epitope that is only accessible in areas of myelin degeneration (Matsuo et al., 1997). Consistent with previous study (Cantoni et al., 2015), we were still able to find a certain amounts of degraded myelin debris in CC compared to normal mice at week 7, when 2 weeks after CPZ has already been withdrawn. However, 3 weeks of EA treatment significantly reduced the existence of myelin debris (Figures 4A,B). To further confirm this result, we performed red oil O staining to detect the presence of lipid-rich myelin debris. Degraded myelin debris tend to display dark-red deposits and clumps in red oil O staining (Weinger et al., 2011). We found that EA treatment for 3 weeks also reduced dark-red clumps as compared to CPZ group at week 7 (Figure 4C). Thus, we confirm that EA treatment promotes clearance of degraded myelin debris, which is beneficial for subsequent axonal remyelination.

### More phagocyte-like microglial cells assemble into CC during EA treatment

Compelling evidence suggests that microglia, especially M2-polarized cells, confer neuroprotection in MS through enhancing clearance and phagocytosis of degraded myelin debris, as well as recruiting OPCs to promote remyelination (Jurevics et al., 2002; Mikita et al., 2011; Vaknin et al., 2011; Olah et al., 2012). In this regard, we reasoned that the decrease of degraded myelin debris and the promotion of remyelination resulting from EA treatment could be related to the regulatory effect of EA on microglia. To prove this hypothesis, microglia in areas of CC were assessed by staining Iba1. The CPZ model is a demyelinating model of non-autoimmune character with intact brain-blood barrier hardly ever allowing large amount of peripheral monocytes/macrophages to infiltrate the CNS (McMahon et al., 2002; Remington et al., 2007). Additionally, previous studies rarely distinguished brain-resident microglia from macrophages of peripheral origin, with the two cell types being phenotypically nearly the same. For this reason, in this study we refer to microglia/macrophages as microglia.

**Figure 5:**
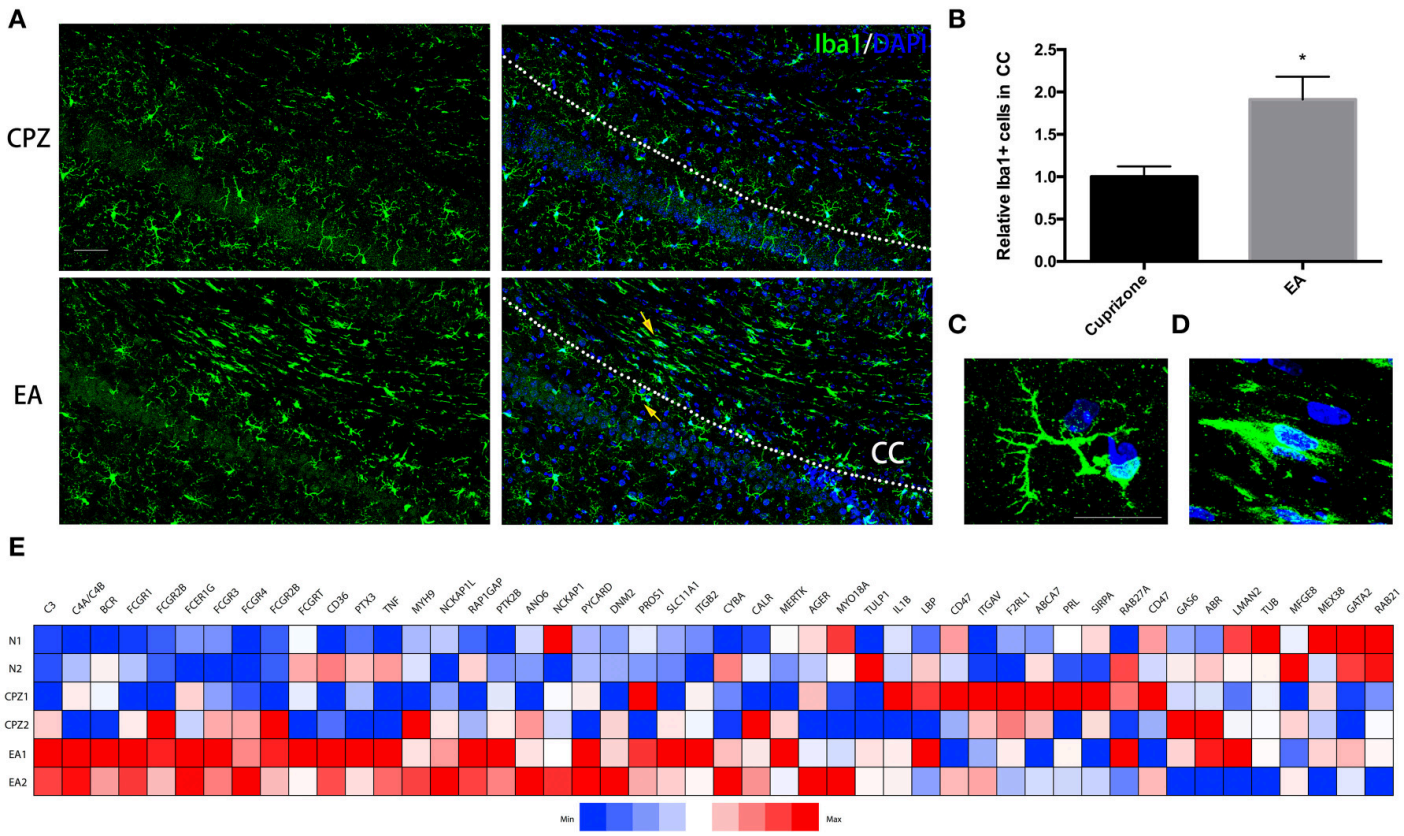
**More microglial cells displaying ameboid and phagocytic shape assemble into corpus callosum during EA treatment. (A)** Microglia assembing into corpus callosum at week 7 was detected by Iba1 (green); nuclei was labeled by Dapi (blue). **(B)** Bar graph shows the semi-quantification of IOD of Iba1 positive cells inside the area of corpus callosum (Data represents the means ± S.E.M.; *n* = 3/group, ^*^p < 0.05 compared to cuprizone group). **(C,D)** Higher magnification of indicated microglia in **(A)**: **(C)** representative microglia outside the corpus callosum with ramified shape displaying more branches; **(D)** representative microglia displaying ameboid and phagocytic shape inside the area of corpus callosum after EA treatment **(E)** A heatmap shows the gene expression of phagocytosis-related genes (according to the gene annotation of “positive regulation of phagocytosis” from AmiGo2; GO: 0050766). Bar = 50 μm for **(A)**; Bar = 20 μm for **(C,D)**.

At week 7, when EA treatment was in its 3rd week, more microglia assembled into CC in EA group than CPZ group (Figures 5A,B). Notably, we found the morphology of microglia assembled into CC of EA group was quite different from that of CPZ group as well as the microglia outside the CC in EA group (Figures 5A,C,D). Microglia in CPZ group and in areas outside the CC of EA group were mostly ramified microglial cells, while microglia assembled into CC of EA group looked more like phagocytic microglia for these cells generally displaying an ameboid, plump shape, indicating an active phagocytic process taking place in CC after EA treatment, which supposedly is the cause for enhanced clearance of myelin debris. To further prove it, we checked the gene expression relating to phagocytosis according to the gene annotation of *Postitive Regulation of Phagocytosis* (GO: 0050766) from AmiGo2 (http://amigo.geneontology.org/amigo/term/GO:0050766). A large number of phagocytosis-related genes were up-regulated after EA treatment (Figure 5E). Taken together, these results prove that EA facilitates microglia assembling into corpus callosum exerting phagocytic function.

### EA facilitates the change of assembled microglia toward M2 phenotype

**Figure 6:**
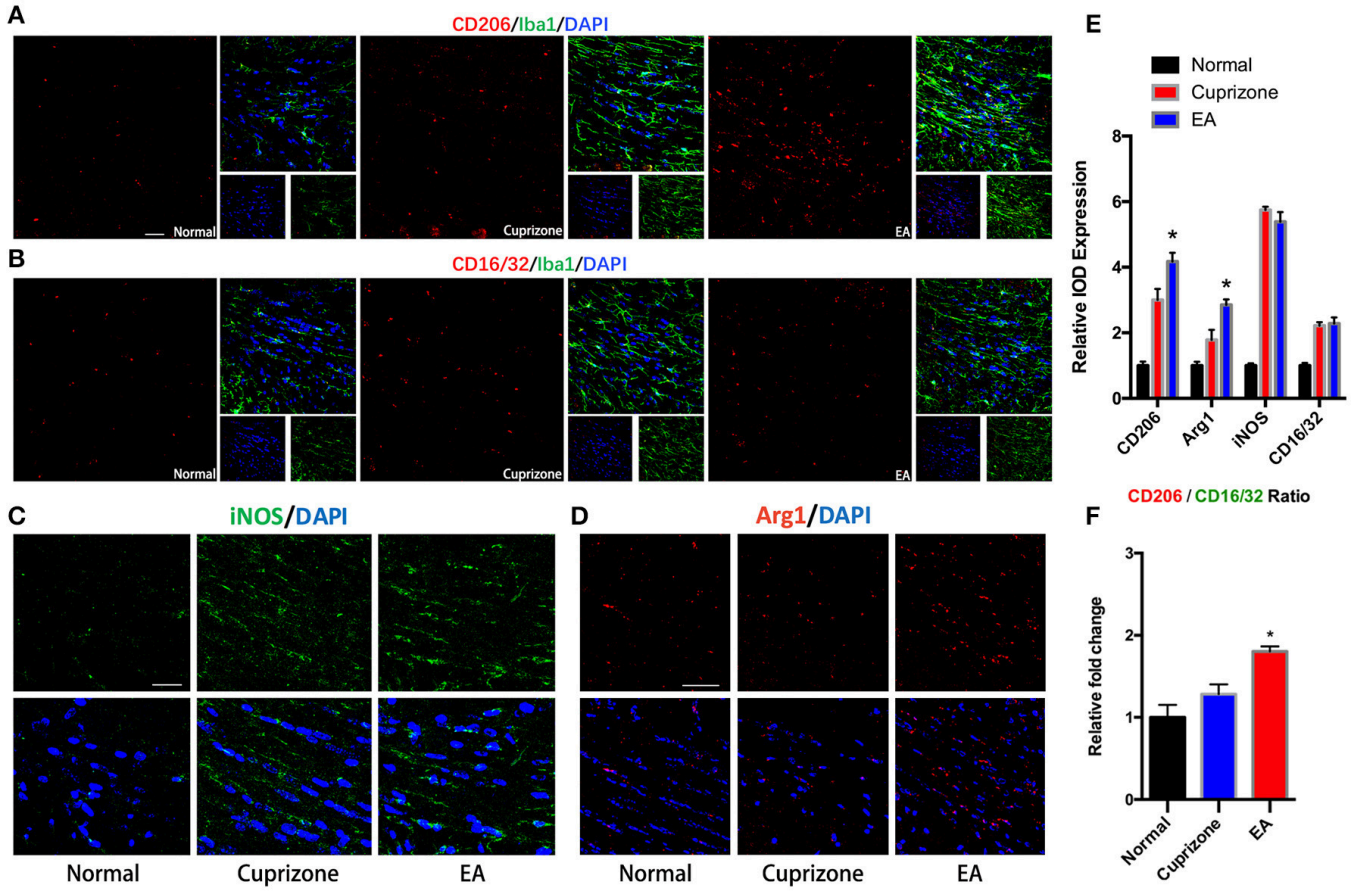
**EA induces higher CD206/CD16/32 ratio in assembled microglia**. **(A)** Immunofluorescence staining of CD206 (red), Iba1 (green), and Dapi (blue). **(B)** Immunofluorescence staining of CD16/32 (red), Iba1 (green), and Dapi (blue). **(C,D)** Immunofluorescence staining of iNOS (green), Arg1 (red), and Dapi (blue). **(E)** Statistical graph showing relative IOD expression of CD206, Arg1, CD16/32, and iNOS in different groups. **(F)** Statistical graph showing relative CD206/CD16/32 ratio in different groups. Bar = 50 μm; data represents the means ± S.E.M. (*n* = 4/group, ^*^p < 0.05 compared to cuprizone group).

It has been reported that microglia tend to change their phenotype toward M2 phenotype during the process of myelin debris clearance, and microglia with M2 property exert protective effect in demyelination (Kotter et al., 2006; Neumann et al., 2008; Skripuletz et al., 2013). Therefore, we conducted immunofluorescence staining to detect expression of markers of M2 and M1 phenotype on microglia in CC. The expression of CD16/32 and iNOS (M1 markers) were elevated in both CPZ group and EA group due to exposure to cuprizone, but there is no difference of their expression between CPZ and EA group (Figures 6B,C,E). On the contrary, we found significantly elevated expression of CD206 and Arg1 (M2 markers) after EA treatment compared to CPZ group (Figures 6B,D,E). Since we observed elevated expression of Iba1 in corpus callosum at week 7 (Figures 5A,B), the increasd expression of M2 markers could also result from the increased number of microglia assembling in CC. So we then compared the CD206/CD16/32 ratio in different groups and we found a higher CD206/CD16/32 ratio after EA treatment. These results indicate that more microglia in corpus callosum change their phenotype to M2 phenotype after EA treatment (Figure 6F).

## Discussion

The goal of the current study was to determine the efficacy of electroacupuncture on remyelination and to explore the underlying mechanisms. In the present study, we demonstrated the following: (1) EA promotes remyelination in CPZ-induced demyelinating model and ameliorates the subdued motor coordination impairments resulting from extensive CNS demyelination; (2) EA significantly eliminates the accumulated degraded myelin debris in CC; (3) the clearance and removal of degraded myelin debris caused by EA is associated with more microglia assembling into the area of CC; (4) microglial cells in CC are mostly ameboid-shaped phagocytic cells with relatively higher M2 property during EA treatment. These results not only prove the efficacy of EA on remyelination, but also elucidate a possible mechanism through which EA regulates the polarization and function of microglia to enhance the clearance of myelin debris.

Seeking feasible methods toward MS treatment is still a long-cherished wish for neurological physicians and neuroscientists globally. Despite much attention having been drawn to immunotherapies in the past, promoting remyelination in MS patients seems more and more crucial because immunomodulatory therapies mainly play a part in reducing immune activity and blocking the infiltration of peripheral immune cells into CNS, but little is done to enhance the process of remyelination (Hartley et al., 2014; Olsen and Akirav, 2015). However, since demyelination is the most critical pathological change of MS patients, which may directly influence the locomotor functions and quality of life, remyelination in lesion areas is indispensable for MS patients. Studies in experimental model of MS have elucidated that preservation of myelin sheath as well as enhancement of axonal remyelination can improve neuronal function and halt the progression of disability (Chari and Blakemore, 2002; Nave, 2010).

The slow progress in finding a practical cure for MS has prompted us to expand our horizon and excavate potential treatment from traditional medicine. Acupuncture is an ancient treating method originating from TCM and is still being widely used worldwide to treat CNS diseases (Zhao, 2008). Although MS is currently not on the list of diseases recommended for acupuncture treatment as assessed by National Institute of Health and World Health Organization, it is still accepted as a complementary therapy with certain curative effect. However, the role of EA in MS and its experimental models remains to be elucidated. Here in our study, we selected two acupoints, Baihui (GV20) and Zhiyang (GV9), to implement EA treatment. The reason why we chose the two acupoints are chiefly as follows: 1. Baihui (GV20) and Zhiyang (GV9) belong to the Governing Vessel (Du meridian), which regulate brain activities and mental disorders according to TCM (Sun et al., 2015; Wu et al., 2015); 2. Anatomically, the connection between this two acupoints runs through cervical cord and brain, covering the demyelinated lesion areas of CPZ fed mice.

The CPZ model we established in our lab exhibited analogous characteristics as previously reported models, which included obvious weight loss during the first 2 weeks, poor performance in beam walking test, and severe demyelination after 5 weeks of CPZ treatment (Skripuletz et al., 2010; Praet et al., 2014). To better test the neurological behaviors, pole test was innovatively introduced to CPZ model and we found that mice with demyelination exhibited defects in turn-back performance, but not in touch-down (descending) performance. It is possible that the behavior of turning in pole test is a coordinative and collaborative motion with the help of hind limbs while the behavior of descending is mainly a motion that requires muscle strength of fore limbs. From our observation, CPZ mice did not exhibit obvious defects in muscle strength compared to the case of EAE mice (Wang et al., 2012), and other studies also reported only mild paralysis mainly in the hind limbs of CPZ treated mice rather than fore limbs (Franco-Pons et al., 2007). Our results in pole test further confirmed this and provided new information on the behavior of CPZ mice.

Mice fed with CPZ showed a peak of sickness at and around week 4, so we treated them from this time point to the day they were sacrificed. We found that successive EA treatments gradually improved the neurological behaviors and promoted recovery of model mice. Nevertheless, we observed some inevitable fluctuations in the results of behavioral tests even in normal mice during the whole scheme of 7 weeks, because there were too many factors that could affect behavioral results, such as individual differences, weight change due to feeding, stressors, learning abilities, etc (Bogdanova et al., 2013). So we further examined the effects of EA by molecular biological techniques and immuno-techniques. The results we obtained indicate that EA treatment enhances the expression of myelin-related components, which further support the results we obtained from behavioral tests. Together with previous research (Ding et al., 2013; Huang et al., 2015; Liu et al., 2015), we are inclined to conclude that EA is an effective healing method to facilitate remyelination in diseases with demyelination. However, we found a disparity in our RT-PCT results: the mRNA expression of MBP (also PLP1) at week 7 is not down-regulated whereas the expression of MBP detected by Western Blot and immuno-staining is reduced. Since cuprizone model is a chronic demyelinating model with spontaneous remyelination, we speculate that the mRNA expression of MBP/PLP1 might revert to normal level at week 7. However, the existence of myelin debris may postone the process of post-transcriptional processing and post-transcriptional translation of protein, and consequently result in delayed protein synthesis of MBP/PLP1. Due to technical difficulties, we did not perform electron microscopy to observe, ultrastructurally, the effect of EA on remyelination, but this will be addressed in our ongoing studies.

Myelin debris is a pathological substance accumulated during CPZ treatment and the process of demyelination in MS patients. Previous studies have demonstrated that the existence of degraded myelin debris is harmful to remyelination and that the speed of CNS remyelination is strongly correlated with the rate of the phagocytosis and clearance of myelin debris (Kotter et al., 2006; Lampron et al., 2015). The emergence of antibodies which can detect degenerated myelin debris (dMBP) is a milestone in the study of demyelinating diseases (Matsuo et al., 1997, 1998). By using antibodies binding degraded myelin debris, we are now able to detect degraded myelin debris and understand the role of myelin debris in demyelinating diseases (Ye et al., 2007; Williams et al., 2014). Though the role of microglia in MS as well as its animal models is an object of dispute, accumulating evidence suggests that microglia play a crucial role in the removal and clearance of degraded myelin debris in CNS (Kotter et al., 2006; Neumann et al., 2008; Rawji and Yong, 2013; Skripuletz et al., 2013). Loss-of-function studies have proved that dysfunction of microglia in demyelinating models will slower the rate of clearance of myelin debris and postpone remyelination process (Kotter et al., 2005; Weinger et al., 2011; Cantoni et al., 2015; Kawabori et al., 2015; Lampron et al., 2015). However, it is still not fully understood, whether higher amount of microglia is beneficial to remyelination. Our results seem to provide the first proof for remyelinating effect of larger population of microglia. Using dMBP antibody, we tentatively made an attempt to detect myelin debris after EA treatment, and much to our surprise, EA exhibited unexpected ability to remove myelin debris. Furthermore, we identified that the myelin clearing effect of EA was mediated by elevated microglia assembling into corpus callosum. RNA-seq result shows that most of the genes relating to positive regulation of phagocytosis are up-regulated during EA treatment. It is worth mentioning that all the Fc receptors in this gene annotation are up-regulated after EA treatment. It's well-known that Fc receptors confer protective function in immune system since they are actively involved in a wide variety of process, mainly phagocytosis. Notably, Fc-gamma receptors are also expressed on cells of oligodendrocyte lineage, and they are reported to control the differentiation cascade of oligodendrocyte that subsequently form myelin sheath, indicating a possible role of Fc receptors in remyelination (Nakahara et al., 2003; Nakahara and Aiso, 2006). In addition to this, it is intriguing that we also found elevated expression of genes of complement system (notably C3, C4). The complement components are highly involved in the process of phagocytosis (Tyler and Boulanger, 2012) and C3/C3R pathway not only participates in the process of microglial phagocytosis but also plays an important role in the priming of microglia (Fu et al., 2012; Ramaglia et al., 2012; Hong et al., 2016). These results, together with plump, ameboid, and phagocyte-like shape of microglia observed after EA treatment, indicate an active process of phagocytosis during EA treatment.

We refer to microglia/macrophages as microglia in our study as we have mentioned above. Microglia of anti-inflammatory M2 phenotype exert lots of protective functions including but not limited to the phagocytosis of myelin debris and the recruitment of OPCs. Here in our study we demonstrate the microglia assembled into CC during EA treatment are more likely to possess M2 property as compared to CPZ group. Since M2 microglial cells drive oligodendrocyte differentiation during CNS remyelination (Miron et al., 2013), this finding, to some extent, is also indicative of a potential effect of EA on the recruitment and differentiation of OPC. However, we did not probe into the effect of EA on OPC in our current study. O4 is a marker in cells of oligodendrocyte lineage that appears early on the stage of OPC differentiation and exists also in mature oligodendrocyte (Schumacher et al., 2012). Though we found elevated expression of O4 after EA treatment at week 7, it cannot be explained as a result of increased OPC differentiation. Following studies focusing on the effect on OPC differentiation during EA treatment may quantify the number of Olig2+/APC+ cells to evaluate differentiation.

Another shortcoming of our current study is that we did not focus on the reaction of astrocyte as well as the crosstalk between astrocyte and microglia. It is reported that in cuprizone model, astrocytes regulate the recruitment of microglia and the process of myelin debris clearance (Skripuletz et al., 2013). We are not quite sure about the role of astrocytes in cuprizone model during EA treatment and our further studies should also pay attention to it.

In a recent study, the research showed that EA can facilitate remyelination via promoting the proliferation of oligodendrocyte and inhibiting its death in SCI model (Huang et al., 2015), and here we report a new mechanism underlying the remyelinating effect of EA, which is by recruiting microglia into areas with demyelinating lesions to clear degraded myelin debris. However, further studies have to be conducted to understand the neural pathway and the precise molecular and cellular mechanisms of how EA regulate microglia recruitment, migration as well as polarization.

## Author contributions

JW, GW, and KZ conceived and designed this research. KZ, JS, ZK, JW, and ZZ conducted the experiments. KZ and JW analyzed the results and data; GW and JS discussed the data; ZK assisted in plotting figures. KZ, JS, ZK, GW, and JW wrote the first draft of this paper; KZ and JS revised the paper; JW, GW, ZK, KZ, JS, and ZZ approved the final version.

## Funding

This work was sponsored by National Science Foundation of China (81202746), the National Key Basic Research Program of China (2013CB531906), the Development Project of Shanghai Peak Disciplines-Integrated Chinese and Western Medicine, and Zhengyi Program (S15-13) of College of Basic Medicine of Fudan University.

### Conflict of interest statement

The authors declare that the research was conducted in the absence of any commercial or financial relationships that could be construed as a potential conflict of interest.

## Supplementary material

Video 1 **Beam walking test (normal)**.

Video 2 **Beam walking test (CPZ)**.

Video 3 **Pole test (normal)**.

Video 4 **Pole test (CPZ)**.

Data sheet 1 **Supplementary figures**.

Data sheet 2 **RNA-seq results**.
