# Supplementary material for: Electroacupuncture Promotes Remyelination after Cuprizone Treatment by Enhancing Myelin Debris Clearance
Source: Front Neurosci. 2017 Jan 10;10:613. doi: 10.3389/fnins.2016.00613 (PMC5222794; doi:10.3389/fnins.2016.00613)
Supplement: Data sheet 1 — Supplementary figures. [file DataSheet1.DOCX]

Supplementary Material

Electroacupuncture promotes remyelination via the recruitment of microglia into corpus callosum clearing myelin debris

**Keying Zhu^1,2,3^, Zheng Kang^1,3^, Zaofeng Zou^1,3^, Jingxian Sun^1,3^, Yan Chen^1,2^, Gencheng Wu^1,2,3^, Jun Wang^1,2,3*^**

*** Correspondence:**

**Jun Wang**，Associate Professor of Neurobiology

Department of Integrative Medicine and Neurobiology

Shanghai Medical College Fudan University P. O. Box 291

138 Yi-Xue-Yuan Road, 200032, Shanghai, China

Telephone: +86-21-54237496

E-mail: [jwangf@shmu.edu.cn](mailto:jwangf@shmu.edu.cn)

## Supplementary Figures


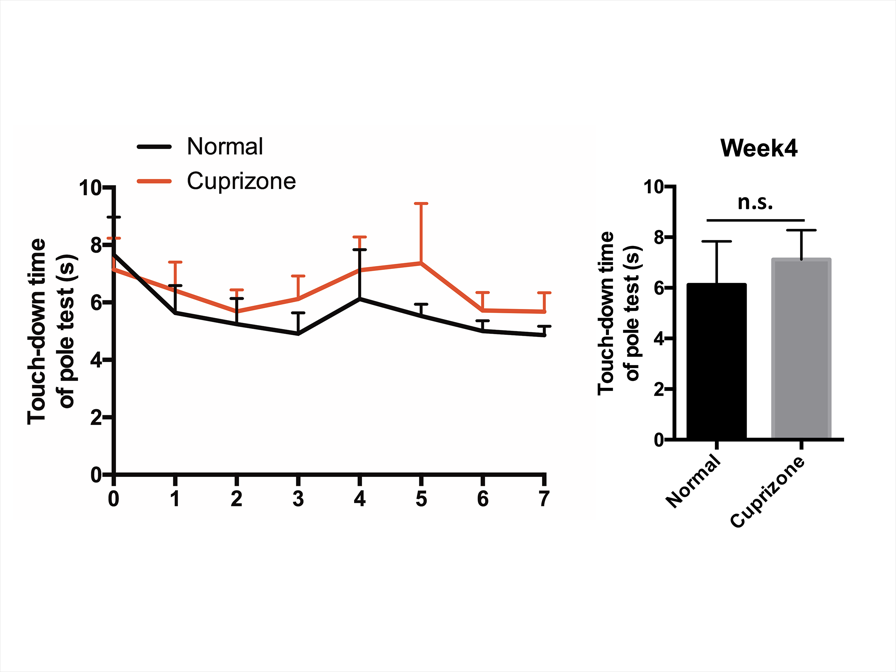


**Figure S1.** **Touch-down time of both groups.** Touch-down time was not affected after CPZ-induced demyelination (n=10 for both groups; n.s., no significance).


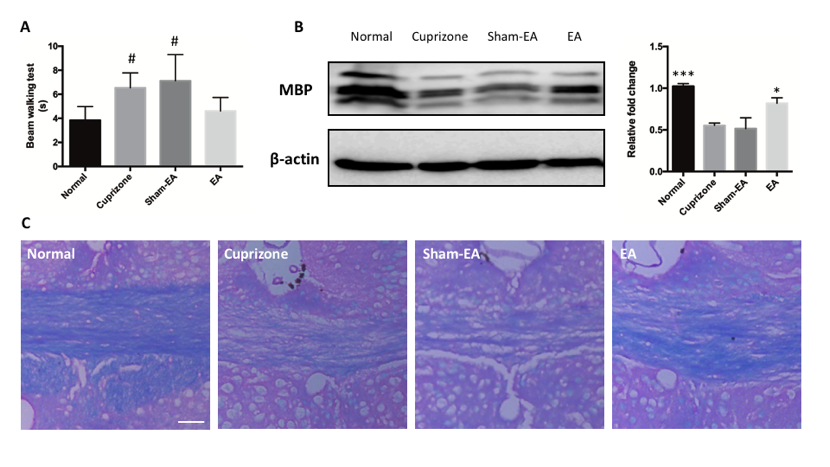


**Figure S2. Sham-EA treatment shows no effect in promoting remyelination. (A)** Reverse time of beam walking test at week 7 (n=6-7 for each group; #p<0.05 compared to normal group). **(B)** The expression of myelin basic protein (MBP) of corpus callosum at week 7 detected by Western Blot and the quantitative data (n=3 for each group; *p<0.05, ***p<0.001 compared to cuprizone group and sham-EA group). **(C)** LFB staining showing pathological change of myelination of corpus callosum at week 7 (scale bar=100μm).
